# Supplementary figures and images for: Membrane Remodeling and Matrix Dispersal Intermediates During Mammalian Acrosomal Exocytosis
Source: Front Cell Dev Biol. 2021 Dec 10;9:765673. doi: 10.3389/fcell.2021.765673 (PMC8708559; doi:10.3389/fcell.2021.765673)

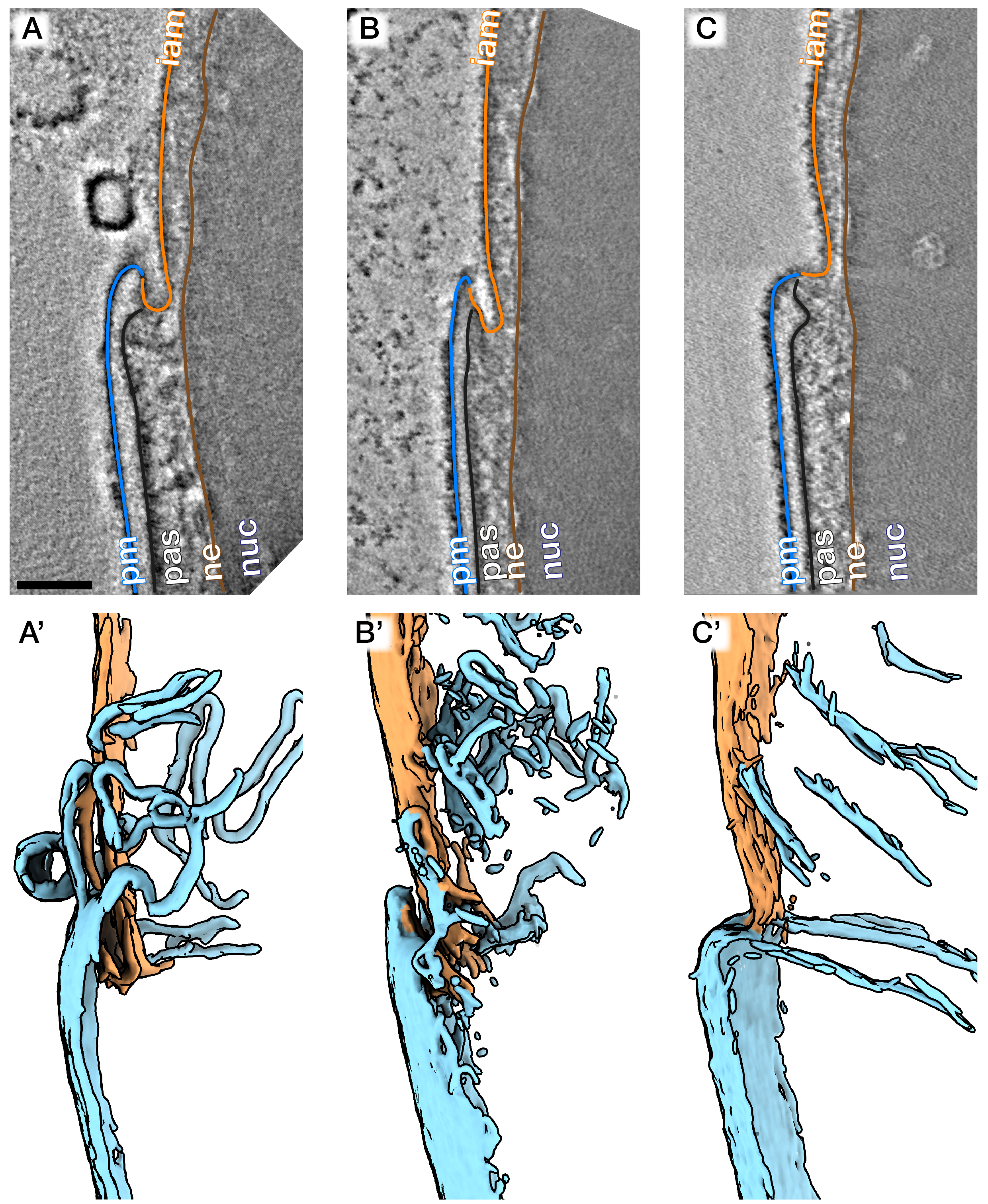

Supplement: Supplementary file 1 [file Image6.tif]

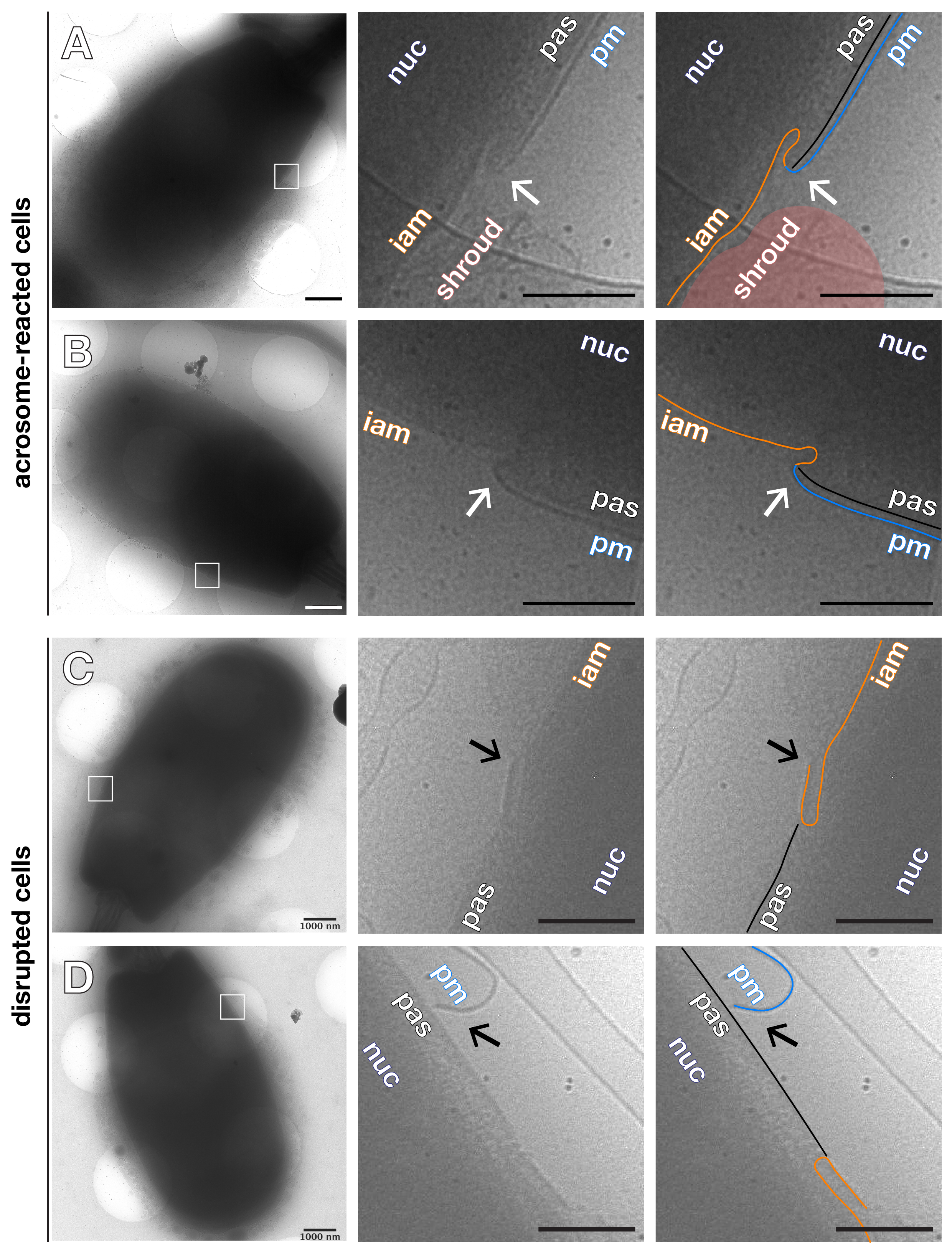

Supplement: Supplementary file 3 [file Image3.tif]

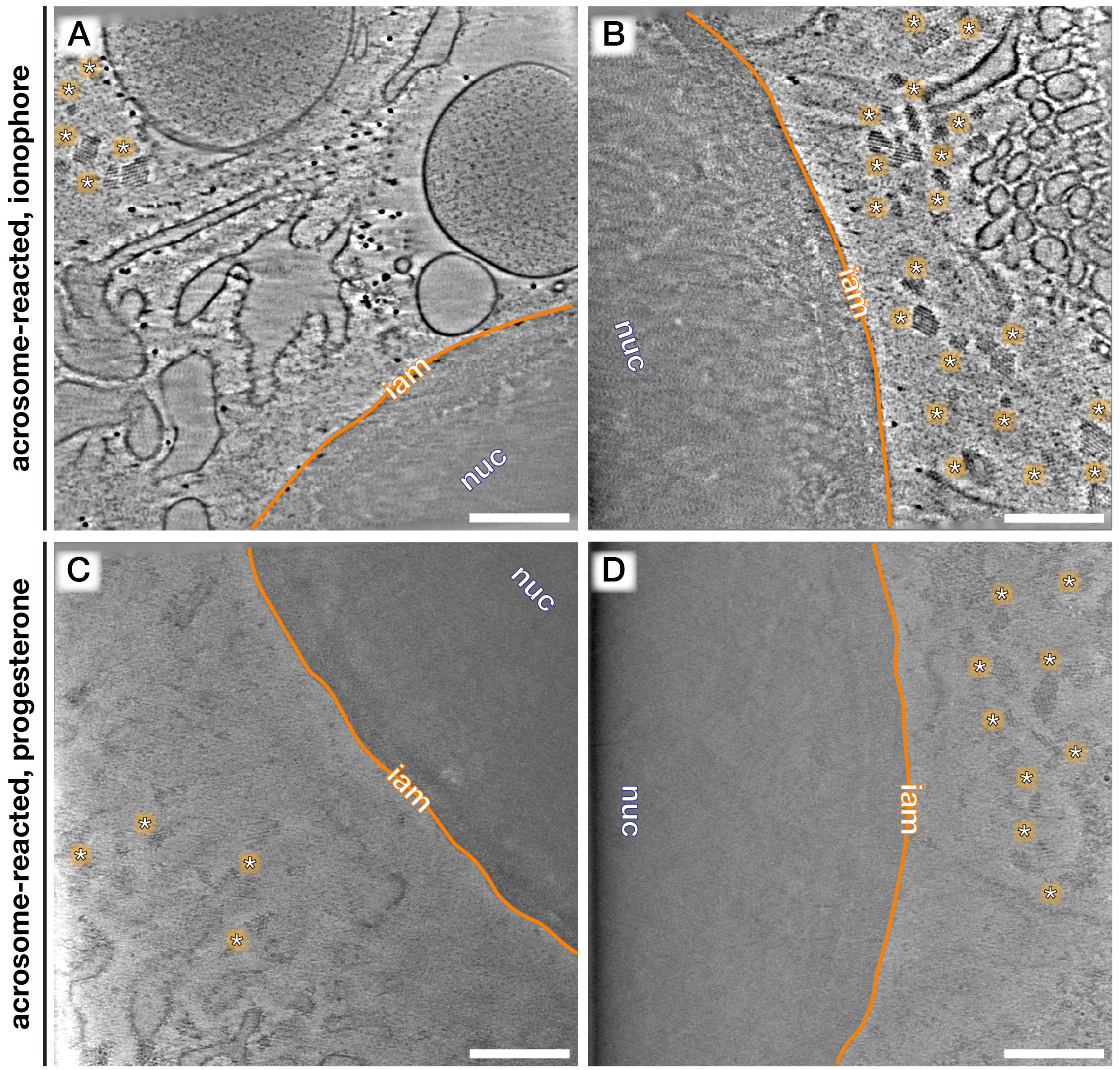

Supplement: Supplementary file 4 [file Image4.tif]

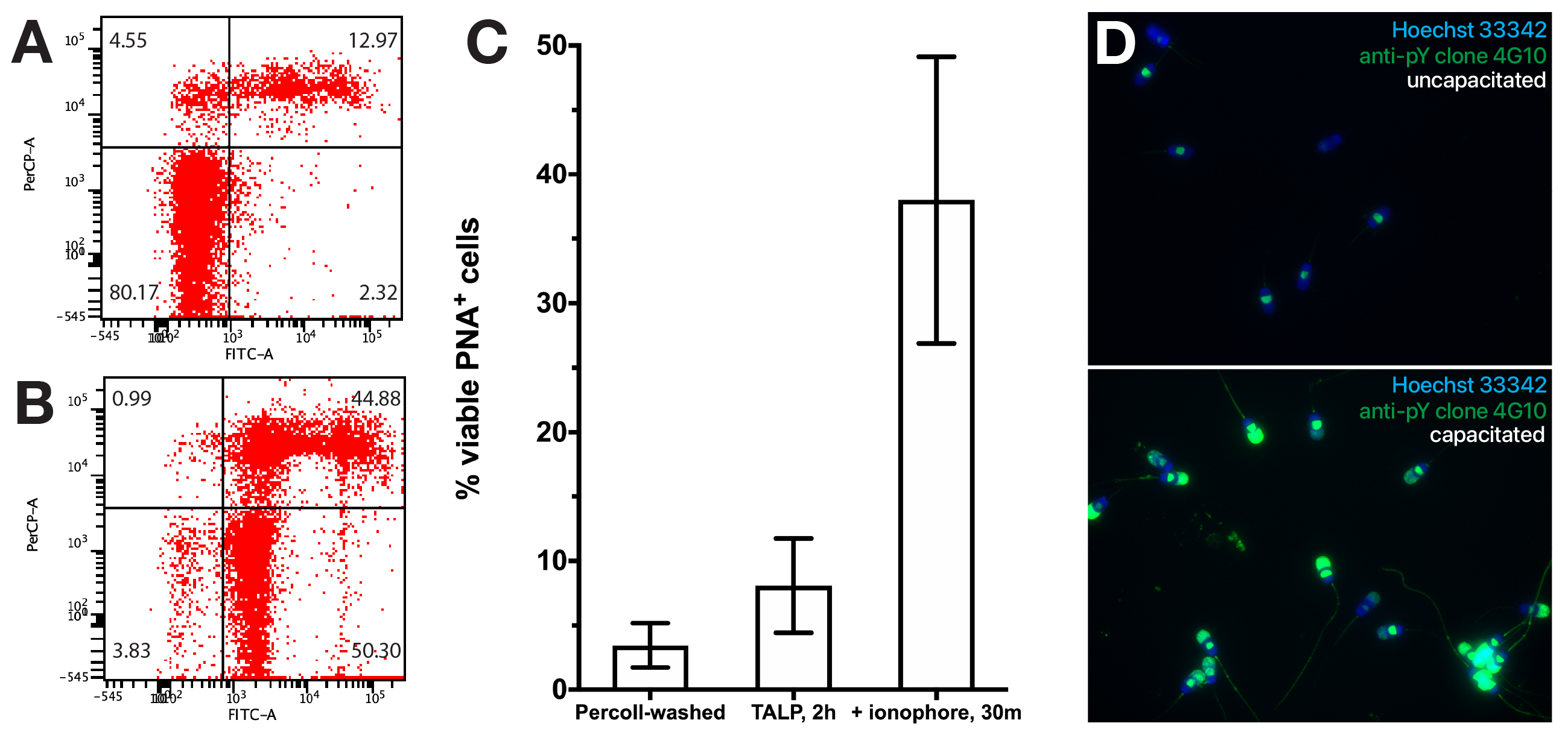

Supplement: Supplementary file 5 [file Image2.tif]

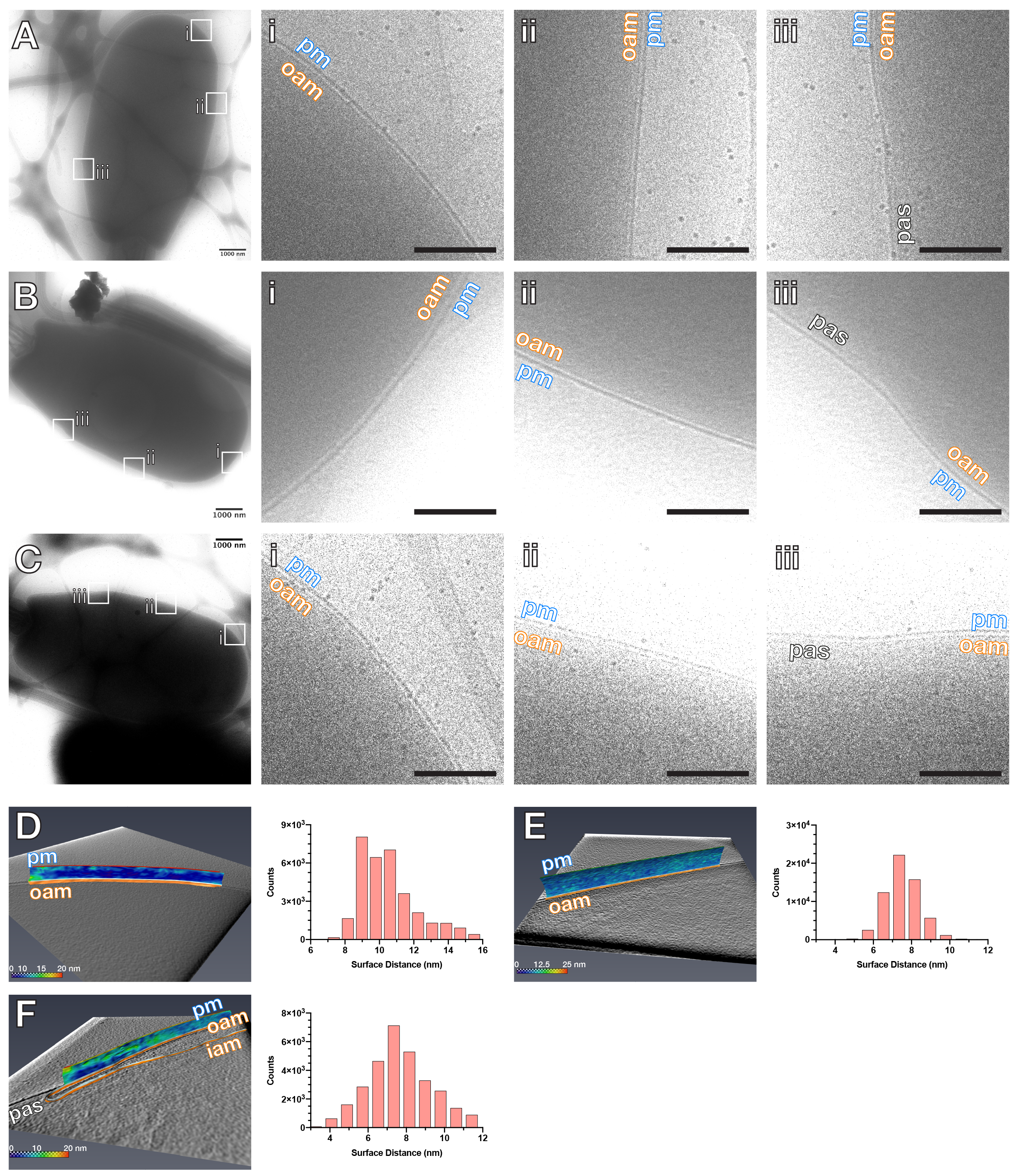

Supplement: Supplementary file 6 [file Image1.tif]

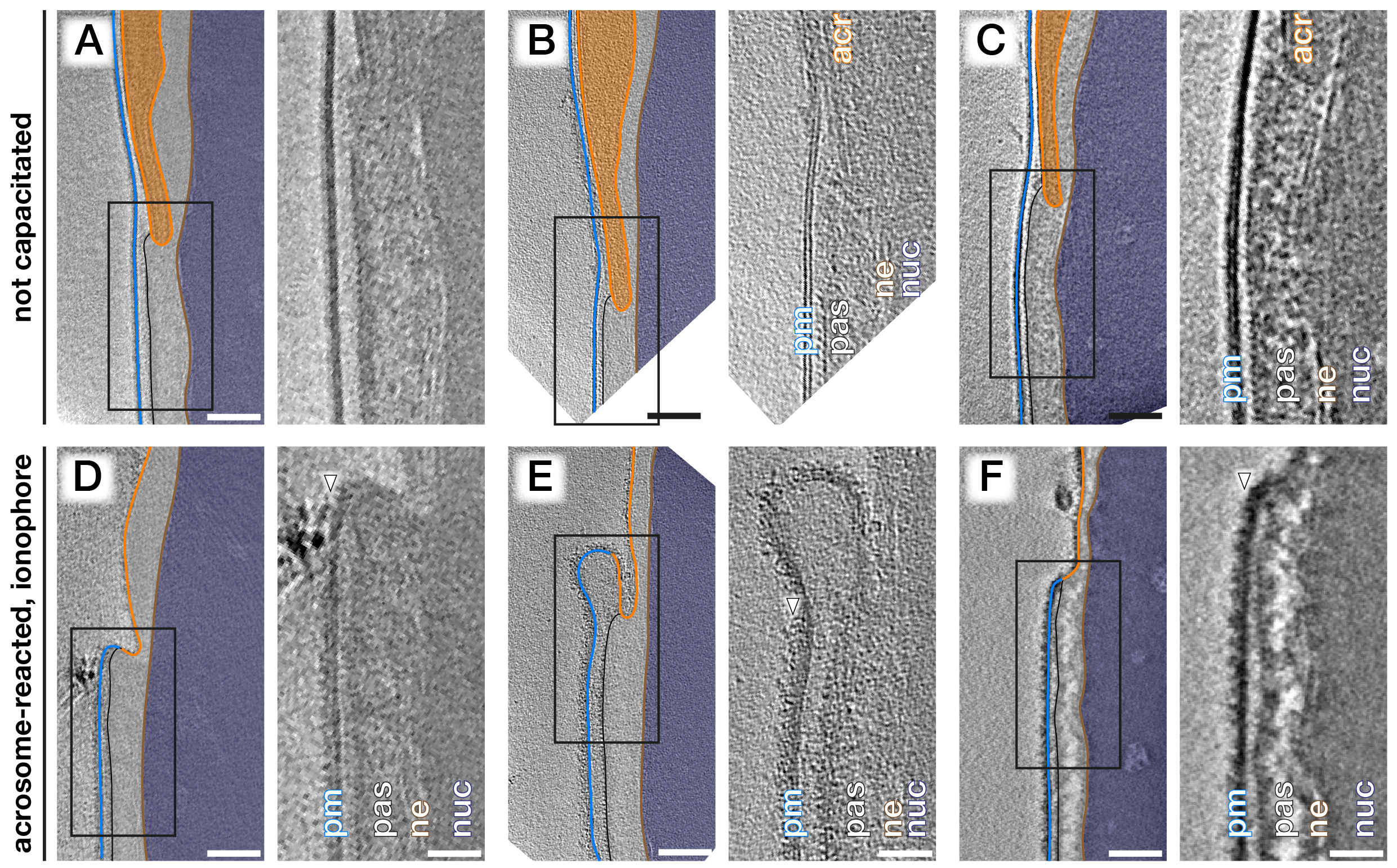

Supplement: Supplementary file 7 [file Image7.tif]

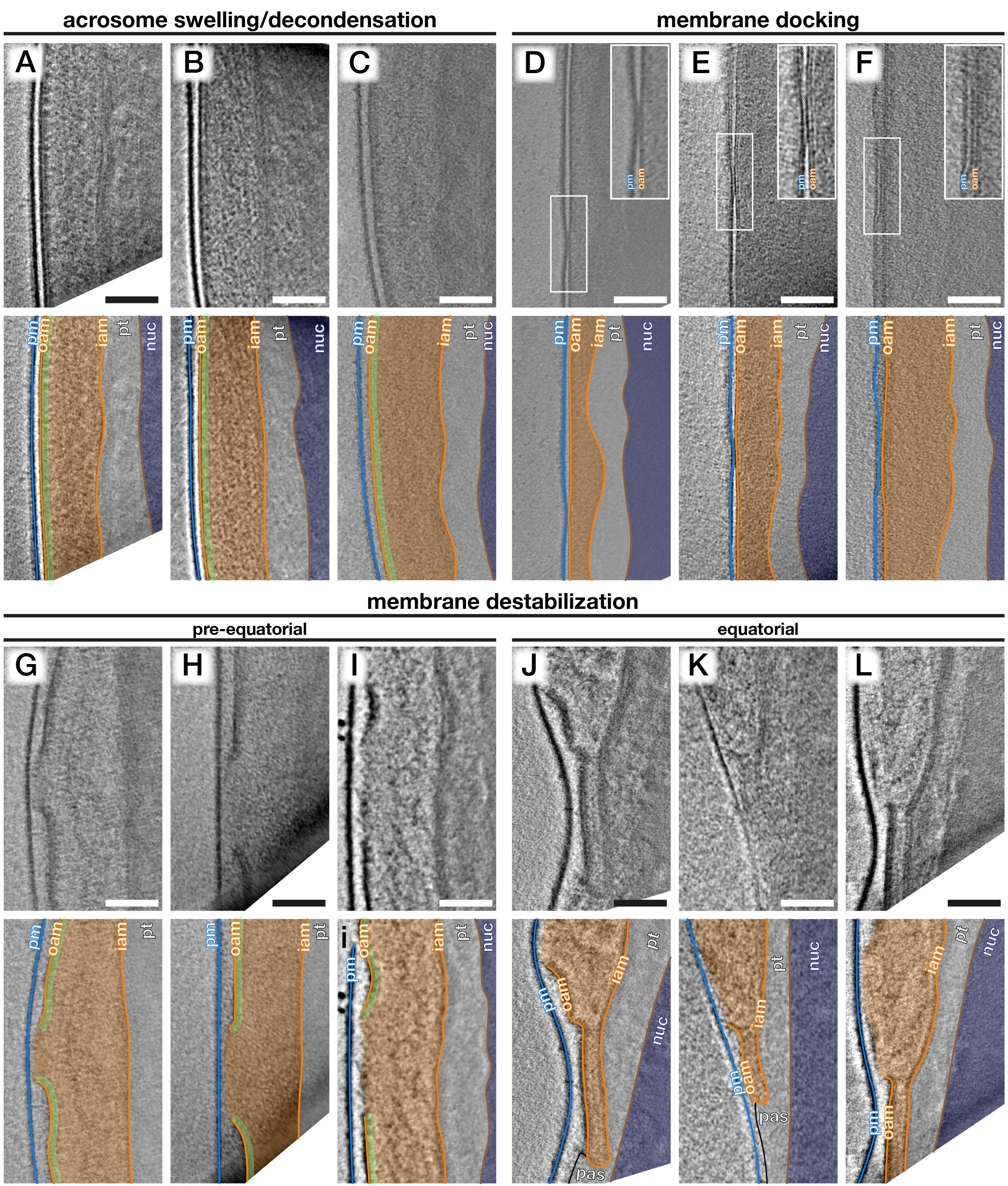

Supplement: Supplementary file 8 [file Image5.tif]
