# Supplementary material for: Membrane Remodeling and Matrix Dispersal Intermediates During Mammalian Acrosomal Exocytosis
Source: Front Cell Dev Biol. 2021 Dec 10;9:765673. doi: 10.3389/fcell.2021.765673 (PMC8708559; doi:10.3389/fcell.2021.765673)
Supplement: Supplementary file 2 [file Table1.docx]

**Table S1.** Dataset summary reporting number of tomograms in the dataset representing each membrane remodelling intermediate.

| **description** | **condition** | | |
| --- | --- | --- | --- |
|  | **uncapacitated** | **capacitated** | **acrosome-reacted** |
| dense acrosome, membranes intact | 26 | 10 | - |
| swollen acrosome, membranes intact | - | 15 | - |
| swollen acrosome, membranes destabilized | - | 16 | - |
| acrosome-reacted, apical region, shroud present | - | - | 13 (ionophore) 5 (progesterone) |
| acrosome-reacted, apical region, shroud absent | - | - | 6 |
| acrosome-reacted, equatorial region | - | - | 37 |
| **total number of tomograms** | **26** | **41** | **56** (ionophore)  **5** (progesterone) |
| **total number of animals** | **7** | **5** | **6** (ionophore) **1** (progesterone) |
